# Comparison of hemodynamics during induction of general anesthesia with remimazolam and target-controlled propofol in middle-aged and elderly patients: a single-center, randomized, controlled trial

**DOI:** 10.1186/s12871-023-01974-9

**Published:** 2023-01-10

**Authors:** Ryo Sekiguchi, Michiko Kinoshita, Ryosuke Kawanishi, Nami Kakuta, Yoko Sakai, Katsuya Tanaka

**Affiliations:** 1 Department of Anesthesiology, Tokushima University Hospital, 2-50-1 Kuramoto-Cho, Tokushima-Shi, Tokushima, 770-8503 Japan; 2 Surgical Center, Tokushima University Hospital, 2-50-1 Kuramoto-Cho, Tokushima-Shi, Tokushima, 770-8503 Japan; 3 Division of Anesthesiology, Tokushima University Hospital, 2-50-1 Kuramoto-Cho, Tokushima-Shi, Tokushima, 770-8503 Japan

**Keywords:** Hemodynamics, Hypotension, Remimazolam, Propofol, Target-controlled infusion

## Abstract

**Background:**

Remimazolam confers a lower risk of hypotension than propofol. However, no studies have compared the efficacy of remimazolam and propofol administered using target-controlled infusion (TCI). This study aimed to investigate hemodynamic effects of remimazolam and target-controlled propofol in middle-aged and elderly patients during the induction of anesthesia.

**Methods:**

Forty adults aged 45–80 years with the American Society of Anesthesiologists Physical Status 1–2 were randomly assigned to remimazolam or propofol group (*n* = 20 each). Patients received either remimazolam (12 mg/kg/h) or propofol (3 μg/mL, TCI), along with remifentanil for inducing anesthesia. We recorded the blood pressure, heart rate (HR), and estimated continuous cardiac output (esCCO) using the pulse wave transit time. The primary outcome was the maximum change in mean arterial pressure (MAP) after induction. Secondary outcomes included changes in HR, cardiac output (CO), and stroke volume (SV).

**Results:**

MAP decreased after induction of anesthesia in both groups, without significant differences between the groups (− 41.1 [16.4] mmHg and − 42.8 [10.8] mmHg in remimazolam and propofol groups, respectively; mean difference: 1.7 [95% confidence interval: − 8.2 to 4.9]; *p* = 0.613). Furthermore, HR, CO, and SV decreased after induction in both groups, without significant differences between the groups. Remimazolam group had significantly shorter time until loss of consciousness than propofol group (1.7 [0.7] min and 3.5 [1.7] min, respectively; *p* < 0.001). However, MAP, HR, CO, and SV were not significantly different between the groups despite adjusting time until loss of consciousness as a covariate. Seven (35%) and 11 (55%) patients in the remimazolam and propofol groups, respectively, experienced hypotension (MAP < 65 mmHg over 2.5 min), without significant differences between the groups (*p* = 0.341).

**Conclusions:**

Hemodynamics were not significantly different between remimazolam and target-controlled propofol groups during induction of anesthesia. Thus, not only the choice but also the dose and usage of anesthetics are important for hemodynamic stability while inducing anesthesia. Clinicians should monitor hypotension while inducing anesthesia with remimazolam as well as propofol.

**Trial registration:**

UMIN-CTR (UMIN000045612).

## Background

Hypotension during general anesthesia is associated with adverse outcomes [1, 2]. Previous studies have suggested that intraoperative hypotension is associated with cardiovascular events and acute kidney injury in patients undergoing non-cardiac surgery [3–5]. Propofol contributes to hypotension during induction of anesthesia, and the risk increases with age [6–9]. Given the risk of perioperative complications in elderly patients [10–12], preventive measures are required against hypotension during general anesthesia.

Remimazolam, an ultra-short-acting benzodiazepine intravenous anesthetic, has an imidazobenzodiazepine skeleton with side chains containing ester bonds in the diazepine ring [13]. Remimazolam potentially has a favorable profile for circulation with a lower risk of hypotension during induction and maintenance of anesthesia than propofol [14–16]. However, previous studies reporting the superiority of remimazolam used bolus doses of 1.5–2.5 mg/kg propofol during induction [14–16], and no study has compared efficacy of remimazolam and propofol administered using target-controlled infusion (TCI). TCI requires a lower dose of propofol to achieve loss of consciousness during induction of anesthesia than manual infusion [17–19]. To verify the superiority of remimazolam over propofol, multiple methods used in clinical practice should be employed. Therefore, this study aimed to compare hemodynamics during induction of anesthesia using remimazolam and target-controlled propofol in middle-aged and elderly patients.

## Methods

This study was reviewed and approved by the Ethics Committee of the Tokushima University Hospital (approval no. 4101). The protocol was registered at the University Hospital Medical Information Network Clinical Trial Registry (UMIN-CTR, UMIN000045612). Prior written informed consents were obtained from all participants. The study complies with the CONSORT statement.

We included 40 patients aged 45–80 years with the American Society of Anesthesiologists Physical Status 1–2 and who underwent surgery under general anesthesia at the Tokushima University Hospital. We excluded patients with the following characteristics: emergency cases, cardiovascular disease, pregnant woman, severe liver dysfunction, dialysis, neurological disorder, intestinal obstruction, drug hypersensitivity, severe lipid metabolism disorder, body mass index ≥ 30 kg/m^2^, or a predicted difficult airway. We also excluded patients who underwent surgeries in the lateral or prone position.

Patients were randomly assigned to the remimazolam or propofol group (*n* = 20 each) by the sealed envelope system. Patients, but not anesthesiologists, were blinded to the group allocation. If the patient was taking antihypertensive drugs regularly, angiotensin receptor blockers (ARBs) were discontinued on the day of surgery, whereas Ca channel blockers were continued. Patients received Ringer solution acetate at 500 mL/h rate through a peripheral venous tract larger than 22G. Remimazolam 12 mg/kg/h or propofol 3 μg/mL (effect site concentration) using TCI system (TERFUSION Syringe Pump Type SS3 TCI, TERUMO Corporation, Tokyo, Japan) was administered along with remifentanil 0.3 μg/kg/min for induction of anesthesia. The TCI pump incorporated the Marsh model [20]. The loss of consciousness was confirmed when the patients failed to respond. Remimazolam was adjusted to 1–2 mg/kg/h and propofol to 2–5 μg/mL by adjusting the bispectral index (BIS) values between 40 and 60 after loss of consciousness. Rocuronium 0.6 mg/kg was administered after loss of consciousness, and endotracheal intubation was performed. After intubation, remifentanil was adjusted to 0.1–0.3 μg/kg/min, and mechanical ventilation was maintained by adjusting the end-tidal CO_2_ between 35 and 45 mmHg. The noninvasive blood pressure was measured using an upper arm cuff every 2.5 min. Cardiac output (CO) and stroke volume (SV) were estimated using pulse wave transit time (estimated continuous cardiac output [esCCO], Nihon Koden, Tokyo, Japan). Hypotension (mean arterial pressure [MAP] < 65 mmHg over 2.5 min) was treated using 4–8 mg ephedrine.

**Fig. 1:**
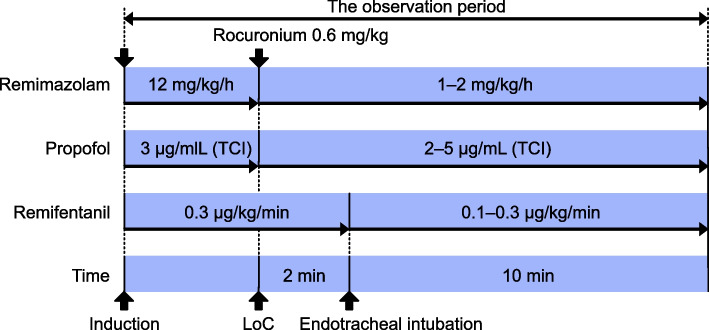
The research protocol. TCI, target-controlled infusion; LoC, loss of consciousness

The primary outcome measure was the maximum change in MAP after induction of anesthesia. Secondary outcome measures were the maximum change in heart rate (HR), CO, and SV. These hemodynamic changes were also examined after adjusting the time until loss of consciousness. Frequency of hypotension (MAP < 65 mmHg over 2.5 min) was also compared. The observation period was from induction of anesthesia to 10 min after endotracheal intubation. Figure 1 shows the research protocol.

### Statistical analysis

The study was designed as a superiority trial, and the sample size was determined as follows: The effect size was set to 1.0 with reference to previous studies that examined the difference in MAP reduction between remimazolam and propofol [15, 16]. After adjusting the α error to 0.05 and power to 0.8, the sample size was calculated to be 34. Therefore, considering a 10% loss of patients, we selected a sample size of 40 patients (*n* = 20 per group).

Data are presented as mean (standard deviation or 95% confidence interval [CI]). Numerical variables between the groups were compared using the Welch’s *t*-test. Analysis of covariance (ANCOVA) was performed to control the effects of covariates. Ratios were compared using the chi-square test or Fisher’s exact test for ≤ 5 cells. All p values were two-sided, and *p* < 0.05 was considered statistically significant. Statistical analyses were performed using EZR (Saitama Medical Center, Jichi Medical University, Saitama, Japan), a graphical user interface for R version 4.0.1 (The R Foundation for Statistical Computing, Vienna, Austria), that provides statistical functions frequently used in biostatistics [21].

## Results

**Fig. 2:**
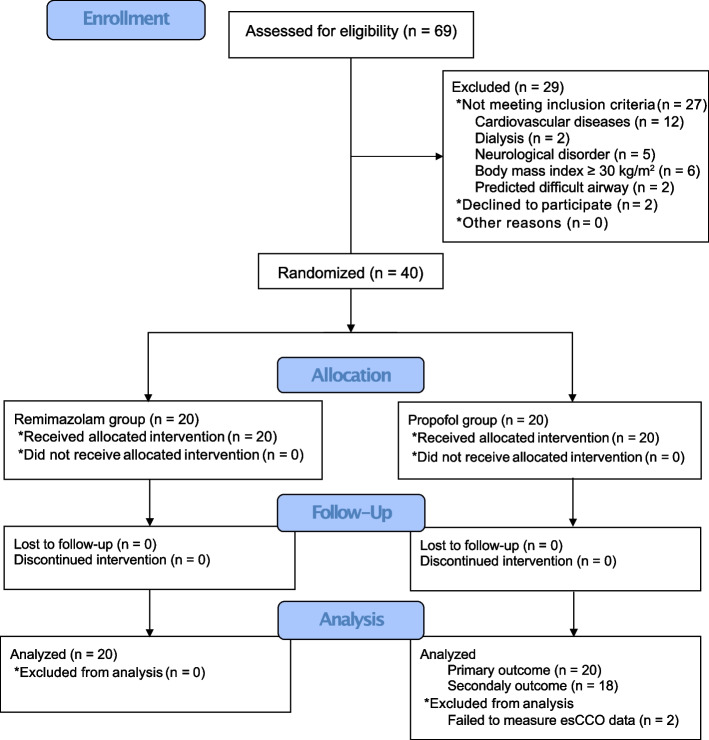
Consort flow diagram. esCCO, estimated continuous cardiac output

This study was conducted at Tokushima University Hospital from November 2021 to February 2022. We initially included 69 patients, but 29 patients who met the exclusion criteria or declined to participate were excluded. Finally, 40 patients were enrolled to the study and randomly assigned to either the remimazolam or propofol group. All patients completed the protocol and were included in the primary endpoint analysis. Two patients in the propofol group were excluded from CO and SV analyses due to lack of esCCO data (Fig. 2).

The characteristics of patients in the groups are listed in Table 1. Eleven (55%) and 6 (30%) patients in the remimazolam and propofol groups, respectively, had antihypertensive drugs, without significant differences between the groups (*p* = 0.201). All patients in the two groups achieved loss of consciousness with the dose of anesthetics specified in the protocol. The mean doses of remimazolam and propofol until loss of consciousness were 0.34 (0.14) mg/kg and 1.21 (0.29) mg/kg, respectively.

**Table 1:** Demographic and clinical characteristics of patients

Characteristics	Remimazolam (*n* = 20)	Propofol (*n* = 20)
**Age, years**	67 (10)	62 (10)
**Sex, male/female**	6/14	4/16
**ASA-PS, 1/2**	5/15	4/16
**Height, cm**	157.8 (5.7)	157.5 (6.0)
**Body weight, kg**	59.2 (8.9)	57.1 (9.4)
**BMI**	23.7 (2.6)	23.1 (3.7)
**Hypertension, + / − **	11/9	7/13
**Antihypertensive drug, + / − **	11/9	6/14
ARBs with short to middle half-life	6	3
ARBs with long half-life	3	0
Ca channel blockers	4	5

Values are mean (standard deviation, SD) or the number of patients

Breakdown of antihypertensive drugs includes duplicates

*n* number, *ASA-PS* American society of anesthesiologists physical status, *BMI* Body mass index, *ARBs* Angiotensin receptor blockers

MAP decreased after induction in both groups, without significant differences between the groups (− 41.1 [16.4] mmHg and − 42.8 [10.8] mmHg in remimazolam and propofol group, respectively; mean difference: 1.7 mmHg [95% CI: − 8.2 to 4.9]; *p* = 0.613). Further, HR, CO, and SV decreased after induction of anesthesia in both groups, without significant differences between the groups (HR: -9.6 [6.6] bpm and − 13.8 [9.9] bpm, *p* = 0.129; CO: − 18.4 [10.8]% and − 24.6 [12.2]%, *p* = 0.101; SV: − 12.7 [8.4]% and − 10.1 [5.7]%, *p* = 0.262; in the remimazolam and propofol groups, respectively) (Table 2).

**Table 2:** Hemodynamic changes after induction

Characteristics	Remimazolam (*n* = 20)	Propofol (*n* = 20)	Mean difference (95% CI)	*p* value
**MAP changes, mmHg**	− 41.1 (9.6)	− 42.8 (10.8)	− 1.7 (− 8.2 to 4.9)	0.613
**HR changes, bpm**	− 9.6 (6.6)	− 13.8 (9.9)	4.2 (− 1.3 to 9.6)	0.129
**CO changes, %**	− 18.4 (10.8)	− 24.5 (12.2)	6.2 (− 1.7 to 13.7)	0.101
**SV changes, %**	− 12.7 (8.4)	− 10.1 (5.7)	− 2.6 (− 7.2 to 2.0)	0.262

Values are the mean (standard deviation, SD)

*n* number, *CI* Confidence interval, *MAP* Mean arterial pressure, *HR* Heart rate, *bpm* Beats per minute, *CO* Cardiac output, *SV* Stroke volume

**Fig. 3:**
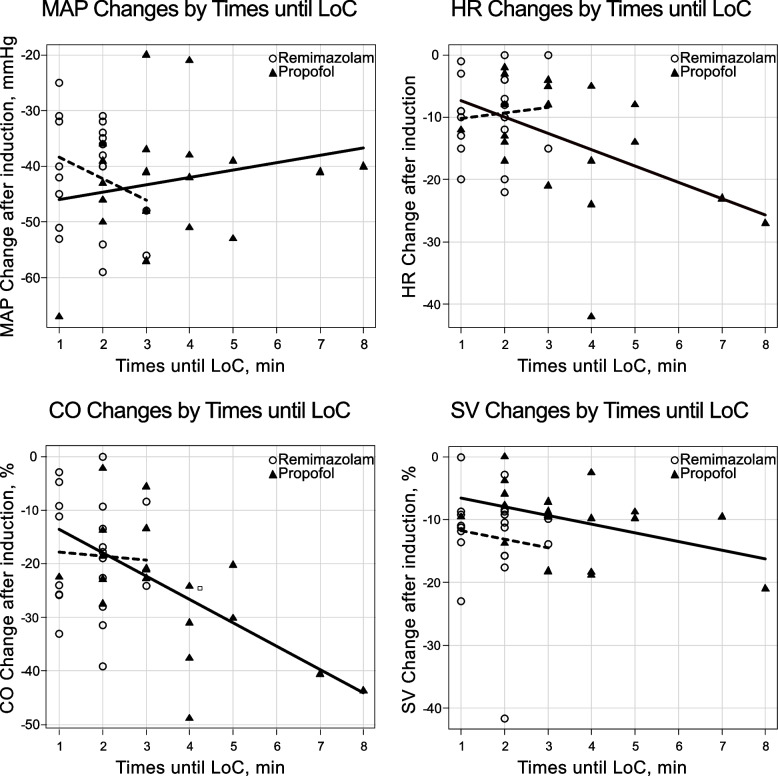
Hemodynamic changes with time until loss of consciousness. LoC, loss of consciousness; MAP, mean arterial pressure; HR, heart rate; CO, cardiac output; SV, stroke volume

The remimazolam group had a significantly shorter time until loss of consciousness than the propofol group (1.7 [0.7] min and 3.5 [1.7] min, respectively; mean difference: 1.8 min [95% CI: 0.8 to 2.6] *p* < 0.001). The time until loss of consciousness had no significant effect on MAP and SV decline (MAP: F[1, 37] = 0.311, *p* = 0.580; SV: F[1, 36] = 2.49, *p* = 0.123), without significant differences between the groups despite adjusting the time until loss of consciousness (MAP: F[1, 37] = 0.535, p = 0.469; SV: F[1, 36] = 3.43, *p* = 0.072). The time until loss of consciousness significantly affected HR and CO decline after induction of anesthesia (HR: F[1, 37] = 5.00, *p* = 0.031; CO: F[1, 36] = 9.17, *p* = 0.005), and the degree of decline increased proportional to time until loss of consciousness. However, changes in HR and CO were not significantly different between the groups despite adjusting time until loss of consciousness (HR: F[1, 37] = 0.011, *p* = 0.916, CO: F[1, 36] = 0.05, *p* = 0.824). According to ANCOVA, the time until loss of consciousness and anesthesia group were not significantly associated for all variables (Table 3, Fig. 3).

**Table 3:** Effects of anesthetic group allocation and time until loss of consciousness on hemodynamic changes after induction of anesthesia

Characteristics	Estimate	Std. error	95% CI	*p* value
**MAP changes, mmHg**				
Intercept	− 42.3	3.1	− 48.7 to − 35.9	< 0.001
Anesthetic group	− 2.8	3.9	− 10.9 to 5.1	0.469
Times until LoC	0.7	1.3	− 1.8 to 3.3	0.580
**HR changes, mmHg**				
Intercept	− 5.9	2.4	− 10.8 to − 0.9	0.021
Anesthetic group	− 0.3	3.1	− 6.5 to 5.9	0.916
Times until LoC	− 2.1	1.0	− 4.2 to 0.2	0.031
**CO changes, %**				
Intercept	− 11.7	3.2	− 18.2 to − 5.3	< 0.001
Anesthetic group	0.9	4.1	− 7.4 to 9.2	0.82
Times until LoC	− 3.9	1.3	− 6.5 to − 1.3	0.005
**SV changes, %**				
Intercept	− 10.4	2.2	− 14.8 to − 6.0	< 0.001
Anesthetic group	5.1	2.8	− 0.5 to 10.7	0.072
Times until LoC	− 1.4	0.9	− 3.2 to 0.4	0.123

*Std* Standard, *CI* Confidence interval, *LoC* Loss of consciousness, *MAP* Mean arterial pressure, *HR* Heart rate, *CO* Cardiac output, *SV* Stroke volume

Seven (35%) and 11 (55%) patients in the remimazolam and propofol groups, respectively, experienced hypotension (MAP < 65 mmHg over 2.5 min), without significant differences between the groups (*p* = 0.341). No postoperative complications related to hypotension, such as myocardial and kidney injuries, were observed in all patients.

## Discussion

The reduction in MAP in the remimazolam and target-controlled propofol groups during induction of anesthesia in middle-aged and elderly patients was not significantly different between the groups, and the mean difference was very small. Moreover, the changes in HR, CO, and SV were not significantly different between the groups. The time until loss of consciousness was significantly shorter in the remimazolam group than in the propofol group. The time until loss of consciousness had no significant effect on the decrease in MAP and SV. A prolonged time until loss of consciousness correlated with greater degree of decline in HR and CO. Despite adjusting the time until loss of consciousness, MAP, HR, CO, and SV were not significantly different between the groups.

Previous studies showed that remimazolam administration for induction or maintenance of anesthesia led to a lesser reduction in blood pressure than propofol [14–16], as opposed to results of the present study. Differences in doses and mode of administration of remimazolam and propofol may influence the results. Doi et al. administered remimazolam (6 or 12 mg/kg/h) or propofol (2.0–2.5 mg/kg, bolus) along with remifentanil for inducing anesthesia [14]. Zhang J et al. administered remimazolam (0.2–0.4 mg/kg, bolus) or propofol (1.5–2.0 mg/kg, bolus), along with sufentanil for inducing anesthesia [15]. Zhang X et al. administered remimazolam (0.2 mg/kg, bolus) or propofol (1.5–2.0 mg/kg, bolus) for inducing anesthesia [16]. In the present study, we administered remimazolam (12 mg/kg/h) or target-controlled propofol (3.0 µg/mL) along with remifentanil for inducing anesthesia. Remimazolam 12 mg/kg/h is equivalent to 0.2 mg/kg/min. As patients required 1.7 (0.7) min to achieve loss of consciousness with remimazolam at 12 mg/kg/h, the dose of remimazolam necessary for loss of consciousness was 0.34 (0.14) mg/kg. Patients required 3.5 (1.7) min to achieve loss of consciousness with propofol at 3.0 µg/mL (TCI), and the dose of propofol until loss of consciousness was 1.21 (0.29) mg/kg; this dose is less than that of previous studies [14–16]. TCI requires a lower dose of propofol to achieve loss of consciousness than manual infusion [17–19]. Remimazolam and propofol decrease blood pressure in a dose-dependent manner [22–25]. The Food and Drug Administration recommends a 1.5 mg/kg maximum dose of propofol for inducing anesthesia in elderly patients [26]. The present study reaffirms that the factors affecting hemodynamics during anesthesia are the dose and usage, as well as the choice of drug.

In the present study, 35% and 55% of patients in the remimazolam and propofol groups, respectively, experienced hypotension (MAP < 65 mmHg over 2.5 min) during induction of anesthesia, although without significant differences between the groups. The number of cases (*n* = 40) in the present study may be low to detect a significant difference in the secondary outcome—192 cases (96 per group) are required to compare the differences between 35 and 55% at α error of 0.05 and power of 0.8. However, even with 192 cases, a significant difference may not be achieved for the degree of MAP reduction (the primary outcome of this study) due to the extremely small mean difference. A previous study reported that MAP below an absolute threshold of 65 mmHg is related to both myocardial and kidney injuries [27]. Thus, hypotension should be closely monitored after induction of anesthesia with both remimazolam and propofol.

Eleven (55%) and 6 (30%) patients in the remimazolam and propofol groups, respectively, had medications for hypertension, without significant differences between the groups. Some antihypertensive drugs are reported to contribute to hypotension during anesthesia [28]. Hojo et al. recently observed that ARBs/angiotensin-converting enzyme inhibitors (ACEIs) with a long half-life and β blockers, but not ARBs/ACEIs with a short–middle half-life and Ca channel blockers, increased the risk of hypotension during anesthesia [29]. Antihypertensive drugs, including ARBs with a long half-life, may have contributed to the hypotension in this study.

We used remimazolam or propofol in combination with remifentanil for induction of anesthesia. Remifentanil may have influenced the results of this study, given its cardiovascular depressant effects [30, 31]. The combination of propofol and remifentanil has been reported to contribute to hypotension during anesthesia [32–34], whereas the data about the interaction of remimazolam and remifentanil is currently lacking. Future studies are needed to determine the cardiovascular effects of the combination of remimazolam and remifentanil.

This study has some limitations. First, we measured non-invasive blood pressure every 2.5 min. More detailed data may have been obtained if arterial blood pressure was continuously measured, such as by radial artery cannulation. However, invasive methods are deemed inappropriate based on patients’ background. Second, for safety reasons, ephedrine was administered to treat persistent hypotension in the patients. The differences between the two groups can be more thoroughly examined if the blood pressure was monitored to its lowest level without treatment, although it is unethical. Third, we used the TCI system for administration of propofol, but not remimazolam. The comparison would have been more appropriate if the same administration method was used; however, administration of remimazolam by the TCI system is not established nor approved in Japan. Remimazolam was continuously administered at 12 mg/kg/h and propofol at 3 μg/mL using the TCI system in this study. Thus, the results may not apply for different doses and drugs. Fourth, the esCCO was used to estimate CO and SV that may differ from the actual values of CO and SV. However, the trending ability of esCCO is clinically acceptable and comparable with the currently available methods using arterial waveform analysis. As esCCO is better at evaluating relative than absolute values, we evaluated CO and SV based on percent changes [35, 36]. Fifth, we set the observation period from induction of anesthesia to 10 min after endotracheal intubation. Previous studies have observed that hypotension is prevalent at 0–10 min after induction of anesthesia or endotracheal intubation [9, 32, 37]. However, a longer observation period may show different results.

## Conclusions

In conclusion, no significant differences in hemodynamics were observed after induction of anesthesia with remimazolam or target-controlled propofol. Thus, not only the choice of drug but also its dosage and usage are important for ensuring hemodynamic stability during induction of anesthesia. Clinicians should carefully monitor hypotension while inducing anesthesia with remimazolam or propofol.

## Data Availability

The datasets used and/or analyzed during the study are available from the corresponding author on reasonable request.

## Abbreviations

TCI: Target-controlled infusion
ARBs: Angiotensin receptor blockers
BIS: Bispectral index
CO: Cardiac output
SV: Stroke volume
esCCO: Estimated continuous cardiac output
MAP: Mean arterial pressure
HR: Heart rate
ANCOVA: Analysis of covariance
CI: Confidence interval
ACEIs: Angiotensin-converting enzyme inhibitors

## References

[1] Monk TG, Bronsert MR, Henderson WG, Mangione MP, Sum-Ping ST, Bentt DR (2015). Association between intraoperative hypotension and hypertension and 30-day postoperative mortality in noncardiac surgery. Anesthesiology.

[2] Sessler DI, Meyhoff CS, Zimmerman NM, Mao G, Leslie K, Vásquez SM (2018). Period-dependent associations between hypotension during and for four days after noncardiac surgery and a composite of myocardial infarction and death: a substudy of the POISE-2 Trial. Anesthesiology.

[3] Walsh M, Devereaux PJ, Garg AX, Kurz A, Turan A, Rodseth RN (2013). Relationship between intraoperative mean arterial pressure and clinical outcomes after noncardiac surgery: toward an empirical definition of hypotension. Anesthesiology.

[4] Roshanov PS, Sheth T, Duceppe E, Tandon V, Bessissow A, Chan MTV (2019). Relationship between perioperative hypotension and perioperative cardiovascular events in patients with coronary artery disease undergoing major noncardiac surgery. Anesthesiology.

[5] Devereaux PJ, Sessler DI (2015). Cardiac complications in patients undergoing major noncardiac surgery. N Engl J Med.

[6] Gauss A, Heinrich H, Wilder-Smith O (1991). Echocardiographic assessment of the haemodynamic effects of propofol: a comparison with etomidate and thiopentone. Anaesthesia.

[7] Yamaura K, Hoka S, Okamoto H, Kandabashi T, Akiyoshi K, Takahashi S (2000). Changes in left ventricular end-diastolic area, end-systolic wall stress, and fractional area change during anesthetic induction with propofol or thiamylal. J Anesth.

[8] El Beheiry H, Mak P (2013). Effects of aging and propofol on the cardiovascular component of the autonomic nervous system. J Clin Anesth.

[9] Reich DL, Hossain S, Krol M, Baez B, Patel P, Bernstein A (2005). Predictors of hypotension after induction of general anesthesia. Anesth Analg.

[10] Polanczyk CA, Marcantonio E, Goldman L, Rohde LE, Orav J, Mangione CM (2001). Impact of age on perioperative complications and length of stay in patients undergoing noncardiac surgery. Ann Intern Med.

[11] van Waes JA, van Klei WA, Wijeysundera DN, van Wolfswinkel L, Lindsay TF, Beattie WS (2016). Association between intraoperative hypotension and myocardial injury after vascular surgery. Anesthesiology.

[12] Griffiths R, Beech F, Brown A, Dhesi J, Foo I, Goodall J (2014). Peri-operative care of the elderly 2014: Association of Anaesthetists of Great Britain and Ireland. Anaesthesia.

[13] Kilpatrick GJ, McIntyre MS, Cox RF, Stafford JA, Pacofsky GJ, Lovell GG (2007). CNS 7056: a novel ultra-short-acting benzodiazepine. Anesthesiology.

[14] Doi M, Morita K, Takeda J, Sakamoto A, Yamakage M, Suzuki T (2020). Efficacy and safety of remimazolam versus propofol for general anesthesia: a multicenter, single-blind, randomized, parallel-group, phase IIb/III trial. J Anesth.

[15] Zhang J, Wang X, Zhang Q, Wang Z, Zhu S (2022). Application effects of remimazolam and propofol on elderly patients undergoing hip replacement. BMC Anesthesiol.

[16] Zhang X, Li S, Liu J (2021). Efficacy and safety of remimazolam besylate versus propofol during hysteroscopy: single-centre randomized controlled trial. BMC Anesthesiol.

[17] Struys M, Versichelen L, Thas O, Herregods L, Rolly G (1997). Comparison of computer-controlled administration of propofol with two manually controlled infusion techniques. Anaesthesia.

[18] Servin FS (1998). TCI compared with manually controlled infusion of propofol: a multicentre study. Anaesthesia.

[19] Hunt-Smith J, Donaghy A, Leslie K, Kluger M, Gunn K, Warwick N (1999). Safety and efficacy of target controlled infusion (Diprifusor) vs manually controlled infusion of propofol for anaesthesia. Anaesth Intensive Care.

[20] Marsh B, White M, Morton N, Kenny G (1991). Pharmacokinetic model driven infusion of propofol in children. Br J Anaesth.

[21] Kanda Y (2013). Investigation of the freely available easy-to-use software ‘EZR’ for medical statistics. Bone Marrow Transplant.

[22] Dai G, Pei L, Duan F, Liao M, Zhang Y, Zhu M (2021). Safety and efficacy of remimazolam compared with propofol in induction of general anesthesia. Minerva Anestesiol.

[23] Schonberger RB, Dai F, Michel G, Vaughn MT, Burg MM, Mathis M (2022). Association of propofol induction dose and severe pre-incision hypotension among surgical patients over age 65. J Clin Anesth.

[24] de Wit F, van Vliet AL, de Wilde RB, Jansen JR, Vuyk J, Aarts LP (2016). The effect of propofol on haemodynamics: cardiac output, venous return, mean systemic filling pressure, and vascular resistances. Br J Anaesth.

[25] Su H, Eleveld DJ, Struys M, Colin PJ (2022). Mechanism-based pharmacodynamic model for propofol haemodynamic effects in healthy volunteers. Br J Anaesth.

[26] FDA (Food and Drug Administration). Highlights of prescribing information. In: The dictionary of FDA-approved Drugs. https://www.accessdata.fda.gov/drugsatfda_docs/label/2022/019627s069lbl.pdf. Accessed 22 Sept 2022.

[27] Salmasi V, Maheshwari K, Yang D, Mascha EJ, Singh A, Sessler DI (2017). Relationship between intraoperative hypotension, defined by either reduction from baseline or absolute thresholds, and acute kidney and myocardial injury after noncardiac surgery: a retrospective cohort analysis. Anesthesiology.

[28] Muluk V, Cohn SL, Whinney C. Perioperative medication management. In: Post TW, Auerbach AD, Holt NF, Givens J, editors. UptoDate. 2021. https://www.uptodate.com/contents/perioperative-medication-management. Accessed 13 Dec 2022.

[29] Hojo T, Kimura Y, Shibuya M, Fujisawa T (2022). Predictors of hypotension during anesthesia induction in patients with hypertension on medication: a retrospective observational study. BMC Anesthesiol.

[30] Elliott P, O’Hare R, Bill KM, Phillips AS, Gibson FM, Mirakhur RK (2000). Severe cardiovascular depression with remifentanil. Anesth Analg.

[31] Joshi GP, Warner DS, Twersky RS, Fleisher LA (2002). A comparison of the remifentanil and fentanyl adverse effect profile in a multicenter phase IV study. J Clin Anesth.

[32] Hino H, Matsuura T, Kihara Y, Tsujikawa S, Mori T, Nishikawa K (2019). Comparison between hemodynamic effects of propofol and thiopental during general anesthesia induction with remifentanil infusion: a double-blind, age-stratified, randomized study. J Anesth.

[33] Poterman M, Scheeren TWL, van der Velde MI, Buisman PL, Allaert S, Struys MMRF (2017). Prophylactic atropine administration attenuates the negative haemodynamic effects of induction of anaesthesia with propofol and high-dose remifentanil: a randomised controlled trial. Eur J Anaesthesiol.

[34] Degoute CS, Ray MJ, Manchon M, Dubreuil C, Banssillon V (2001). Remifentanil and controlled hypotension; comparison with nitroprusside or esmolol during tympanoplasty. Can J Anaesth.

[35] Terada T, Oiwa A, Maemura Y, Robert S, Kessoku S, Ochiai R (2016). Comparison of the ability of two continuous cardiac output monitors to measure trends in cardiac output: estimated continuous cardiac output measured by modified pulse wave transit time and an arterial pulse contour-based cardiac output device. J Clin Monit Comput.

[36] Terada T, Ochiai R (2021). Comparison of the ability of two continuous cardiac output monitors to detect stroke volume index: estimated continuous cardiac output estimated by modified pulse wave transit time and measured by an arterial pulse contour-based cardiac output device. Technol Health Care.

[37] Jor O, Maca J, Koutna J, Gemrotova M, Vymazal T, Litschmannova M (2018). Hypotension after induction of general anesthesia: occurrence, risk factors, and therapy. a prospective multicentre observational study. J Anesth.

